# Converging Resting State Networks Unravels Potential Remote Effects of Transcranial Magnetic Stimulation for Major Depression

**DOI:** 10.3389/fpsyt.2020.00836

**Published:** 2020-08-20

**Authors:** Takuya Ishida, Thomas Dierks, Werner Strik, Yosuke Morishima

**Affiliations:** ^1^Center for Evolutionary Cognitive Sciences, Graduate School of Arts and Sciences, The University of Tokyo, Meguro-ku, Japan; ^2^Department of Neuropsychiatry, Graduate School of Wakayama Medical University, Kimiidera, Japan; ^3^Division of Systems Neuroscience of Psychopathology, Translational Research Centre, University Hospital of Psychiatry, University of Bern, Bern, Switzerland; ^4^University Hospital of Psychiatry, University of Bern, Bern, Switzerland

**Keywords:** major depressive disorder, resting-state functional magnetic resonance imaging (fMRI), functional connectivity, repetitive transcranial magnetic stimulation (rTMS), dynamic causal modeling (DCM), Granger causality analysis (GCA)

## Abstract

Despite being a commonly used protocol to treat major depressive disorder (MDD), the underlying mechanism of repetitive transcranial magnetic stimulation (rTMS) on dorsolateral prefrontal cortex (DLPFC) remains unclear. In the current study, we investigated the resting-state fMRI data of 100 healthy subjects by exploring three overlapping functional networks associated with the psychopathologically MDD-related areas (the nucleus accumbens, amygdala, and ventromedial prefrontal cortex). Our results showed that these networks converged at the bilateral DLPFC, which suggested that rTMS over DLPFC might improve MDD by remotely modulating the MDD-related areas synergistically. Additionally, they functionally converged at the DMPFC and bilateral insula which are known to be associated with MDD. These two areas could also be potential targets for rTMS treatment. Dynamic causal modelling (DCM) and Granger causality analysis (GCA) revealed that all pairwise connections among bilateral DLPFC, DMPFC, bilateral insula, and three psychopathologically MDD-related areas contained significant causality. The DCM results also suggested that most of the functional interactions between MDD-related areas and bilateral DLPFC, DMPFC, and bilateral insula can predominantly be explained by the effective connectivity from the psychopathologically MDD-related areas to the rTMS stimulation sites. Finally, we found the conventional functional connectivity to be a more representative measure to obtain connectivity parameters compared to GCA and DCM analysis. Our research helped inspecting the convergence of the functional networks related to a psychiatry disorder. The results identified potential targets for brain stimulation treatment and contributed to the optimization of patient-specific brain stimulation protocols.

## Introduction

Repetitive transcranial magnetic stimulation (rTMS) is a non-invasive brain stimulation technique to modulate the neural plasticity of the brain. Recent systematic reviews and meta-analyses revealed that rTMS, with high frequency (HF) on left dorsolateral prefrontal cortex (DLPFC) or low frequency (LF) on right DLPFC, is an effective method to treat major depressive disorder (MDD) (1–7). It is frequently recommended that MDD patients who fail their antidepressant treatment be switched to rTMS treatment. However, despite the increasing recognition of rTMS as a potent treatment for MDD, the neural mechanism underlying such success is still elusive.

MDD is a chronic mental illness characterised by multiple cognitive, behavioral and psychological symptoms associated with emotional, cogitative, and motivational impairments, with anhedonia (e.g., loss of energy and motivation, inhibition of thought process) being one of the key symptoms (8, 9). It is also well known that MDD has a high comorbidity with anxiety disorders (10). Traditionally, the symptomatology of MDD is associated with dysfunction of the neural circuits which mediate reward, emotion, and decision making, involving the ventral tegmental area, nucleus accumbens (NAC), amygdala (AMY), and ventromedial prefrontal cortex (VMPFC) (11, 12), an idea supported by neuroimaging findings. The known functions of the DLPFC, however, are not directly related to the core cognitive impairments found in MDD patients such as anhedonia, anxiety, and agitation (13–15). Therefore, despite the associations found between hypoactivity of the DLPFC and MDD (16–18) and between bilateral lesion of the DLPFC and an increased vulnerability to MDD (19), it is premature to argue that DLPFC abnormalities directly leads to MDD symptoms. Considered the absence of direct linkage between the DLPFC and core features of MDD, the positive influence of rTMS treatment on MDD patients is unlikely to be solely explained by modulations in DLPFC activities.

The neurophysiological effects of rTMS are not restricted to the site of stimulation but can be spread to its functionally connected brain areas (20–26). This suggests that the treatment effects of rTMS can also be explained by remote effects, which are also known as network effects. Therefore, the analysis of functional brain networks may unveil the underlying mechanism of rTMS treatment for MDD (21, 27–30). Several studies proposed that the resting-state functional magnetic resonance imaging (fMRI) data of the healthy control (HC) can be used to determine the optimal stimulation site of rTMS treatment for MDD (31, 32). Furthermore, linking the psychopathological characteristics to certain brain regions, which are identified by their known functions or associated neural circuits, have been considered as an important approach to redefine the diagnostic system of psychiatry disorders (33–36). Therefore, we have investigated the functional interactions of HC subjects between rTMS stimulation sites and regions associated with MDD symptoms, aiming to capture clues of the potential mechanisms of rTMS treatment for MDD. To be more specific, we have focused on the NAC, AMY and VMPFC because the functional specializations of these areas are associated with the core clinical symptoms of MDD, lack of motivation, anxiety, and retardation to make decision, respectively. Recent popular approaches to study the directional interactions of brain regions include dynamic causal modeling (DCM) and Granger causality analysis (GCA). DCM, a technique designed to investigate the causal inter-regional interactions among brain networks, has been used to study both normal and aberrant brain network interactions among HC (37–39) and patients suffered from psychiatric disorder (40–42). DCM provides us more detailed information about the functional interactions of brain networks that could not be obtained by the functional connectivity (FC) or conventional regional activation analysis (43, 44). GCA focuses on identifying the directed causal interactions based on time series analysis of precedence and predictability (45) and has been widely applied to fMRI data (46–48).

In the current study, we investigated the intrinsic brain interactions aiming to understand the neurophysiological mechanism of rTMS treatment for MDD. More specifically, we examined whether the mechanism of rTMS treatment effects for MDD could be explained by the inferred remote effects using FC analysis. To this end, we used resting-state fMRI data of a healthy population to study the FC of the brain areas implicated in MDD. We first investigated how the brain areas associated with symptomatology of MDD, namely, the NAC, AMY, and VMPFC, were functionally connected to the bilateral DLPFC—the widely accepted stimulation sites of rTMS treatment for MDD. Next, we performed conjunction analysis and found these three areas converged at the DLPFC as well as at the dorsomedial prefrontal cortex (DMPFC) and bilateral insula, which are considered as potential target for rTMS treatment of MDD. Further, we performed the DCM analysis (49, 50) and GCA (45, 47) to investigate the direction of interactions among the psychopathologically MDD-related areas and the bilateral DLPFC, DMPFC and bilateral insula across the subjects. Finally, we compared the connectivity parameters obtained from FC analysis, DCM, and GCA.

## Materials and Methods

### Data and Image Preprocessing

The resting-state fMRI data of 100 healthy unrelated subjects from the Human Connectome Project (HCP) were used in the current study (51). All data were acquired by customized Siemens 3.0 Tesla Skyra scanners, using a multiband accelerated pulse sequence [number of volume, 1200 (14.33 min); TR, 720 ms; TE, 33.1ms; FOV, 208 × 180 mm; Matrix, 104 × 90; Slice thickness, 2.0 mm; Multiband factor, 8; phase encoding direction R/L].

The “extensively artifact removed” data set pre-processed by HCP were selected. The pipeline of pre-processing included the correction of spatial distortions due to magnetic inhomogeneity, the realignment of head motion, the coregistration to structural MRI data, and reduction of the bias field, normalization to the MNI space, and resampling into 2-mm isotropic voxels. Then global intensity of the entire 4D data set was normalized by a single scaling factor, and non-brain voxels were masked out. The data were further de-noised using the FIX approach (52, 53). Using independent component analysis, components representing the effects of motion, non-neuronal physiological, scanner-related artifacts and other nuisance sources, were subtracted from the original data. Additionally, the head motion-related components [the six rigid-body parameter timeseries, their backward-looking temporal derivatives, plus all 12 resulting regressors squared, as suggested by (54)] were regressed out from the data. More details of the data acquisition protocol and pre-processing pipelines of the current data set can be found in previous studies (55, 56).

Next, we applied spatial smoothing to the “extensively artifact removed” data with a 6-mm full width at half maximum Gaussian kernel using SPM12 (http://www.fil.ion.ucl.ac.uk/spm/software/). After extracting the BOLD time series from the spatially smoothed data for each voxel, temporal band-pass filter (0.009–0.08 Hz) was applied to reduce the LF drift and HF noise (57). Signals correlated with the cerebrospinal fluid (CSF), white matter (WM), and gray matter (GM) were removed from the data by linear regression.

### ROI Specification for Seed-Based FC Analyses

The seed mask for the bilateral AMY was created from the computational anatomy toolbox (CAT12: http://www.neuro.uni-jena.de/cat/) for SPM. To ensure that the seed region of AMY did not include the anterior hippocampus region, we used the probabilistic atlas and created the masks for AMY and anterior hippocampus, with the threshold of both masks set as 0.3. Regions with a larger probability values for anterior hippocampus than for AMY were removed from the AMY mask. Based on previous studies (58–63), the ROI for the bilateral NAC comprised of 8-mm radius of spherical ROIs centered at [x y z] = [14, 10, 0] and [−14, 10, 0] (mm in the MNI coordinate). For the VMPFC, the ROI was specified as 10-mm radius of a spherical ROI centered at [x y z] = [0, 46, −6], which was defined based on previous reports about VMPFC’s involvement in decision making (60–62, 64, 65).

### Seed-Based FC Analysis and Conjunction Analysis for Group Statistics

First, for each ROI, we extracted time course of all voxels included in the ROI, and averaged across all voxels in the ROI. To create a whole-brain voxel-wise correlation map, we calculated the Person’s correlation coefficients between the time course of a ROI and that of all voxels in GM. These correlation coefficient maps were transformed into z score maps using Fisher’s *r*-to-*z* transformation. Finally, the z-transformed correlation maps were subjected to group-level analysis. One-sample t-tests for each seed ROI was performed by setting a threshold at *p* < (0.05/3) with Bonferroni correction, voxel-wise family-wise error (FWE) corrected.

To study the convergence of psychopathologically relevant brain networks, positive correlation map of the NAC, negative correlation map of the AMY, and negative correlation map of the VMPFC were used. Conjunction analysis was conducted to identify the overlap of the three networks and found that they converged at the bilateral DLPFC, dorsomedial prefrontal cortex (DMPFC) and bilateral insula. The overlap regions were considered to be statistically significant at a threshold of *p* < 0.05, voxel-wise FWE corrected.

To evaluate the effect of global signal scaling on the correlational relationships among the brain regions of our interests, the average time courses of the eight ROIs (the AMY, NAC, VMPFC, bilateral DLPFC, DMPFC, and bilateral insula) were calculated for each subject with or without regressing out of the whole GM signal time series, and pairwise correlation coefficients were calculated.

### Granger Causality Analysis

Furthermore, we performed GCA to investigate the effective connectivity among the bilateral DLPFC, DMPFC, bilateral insula, and the three psychopathologically MDD-related areas using the multivariate Granger causality (MVGC) MATLAB toolbox (64). In GCA, a variable X is considered to “Granger cause” a variable Y if the information of the past X helps predicting the future of Y with better accuracy than the predictability of Y itself (45).

The ROIs for the AMY, NAC, and VMPFC were the same as those used in the FC analysis while the ROIs for the DLPFC, DMPFC, and insula were specified by the 8-mm radius of a spherical ROI centered at the peak coordinate adopted from the conjunction analysis (right DLPFC [x, y, z] = [40, 36, 34]; left DLPFC [−36, 44, 28]; DMPFC [4, 18, 46]; right insula [46, 18, 2]; left insula [−40, 16, 4]).

We extracted the average time courses of the eight ROIs for each subject after regressing out the signals related to WM and CSF without band-pass filtering. The MVGC toolbox fit a vector autoregressive (VAR) model to 20 model orders and selected the minimum model order (temporal lag) of two points according to Akaike information criteria. We adopted the model selection rather than fixed length of lag (i.e., 1TR) approach because while TR could vary among data sets, the expected time lag of neural interactions between brain areas remained stable. By using the MVGC toolbox, a corresponding VAR model was estimated, and the autocovariance sequence and time-domain conditional Granger causality were calculated. The causalities for significance were also tested using the mvgc_pval function implemented in this toolbox with the alpha set at 0.05, corrected for false discovery rate (FDR). It should be noted that the Granger causality values must be zero or greater than zero since they are defined by the log-likelihood ratio between the residuals covariance matrix of the VAR models (64).

The mean values of each connectivity parameter across the subjects were calculated (**Table 4**). As the ability to handle large numbers of sources for regions is facilitated by GCA, these results were used to narrow down model space for DCM (65).

### Dynamic Causal Modeling

Dynamic causal modeling (DCM) (66) was applied to examine the influences of the bilateral DLPFC, DMPFC, and bilateral insula on the three depression-related areas. DCM is a Bayesian framework to infer effective connectivity between brain regions in a neural system of interest. Effective connectivity quantifies the directional causal relationship from one area to another (67). We employed the spectral DCM implemented in SPM12 for resting-state fMRI data. Spectral DCM models the cross correlation function of the time series of neuronal fluctuations (spectral densities over frequencies), which makes it more computationally efficient as the estimation of neuronal hidden states is not needed (68). Furthermore, it is more sensitive to group differences for the estimation of effective connectivity parameters (68).

The ROIs of the AMY, NAC, VMPFC, DLPFC, DMPFC, and insula were set as the same as the GCA analysis. To extract the BOLD time series of the eight ROIs for the DCM analysis, we first estimated a GLM including WM and CSF signal time series with a high-pass filter (cut-off frequency = 128 s), then regressed out the nuisance covariates and calculated the eigenvariate time courses.

We specified a fully connected model that has bidirectional connections between any pair of ROIs for each subject because the GCA showed significant Granger causalities for all connections. As the fully connected model contained 8 ROIs, we estimated 64 free parameters which included all possible 56 pairwise connections and 8 self-connections. Then, we performed Bayesian model selection (BMS) using a *post hoc* optimization method to determine the best-fitting model with the best balance between accuracy and complexity (69–71). The corresponding effective connectivity parameters for the best fitting model were then estimated. We also estimated the pairwise FC parameters among the 8 ROIs by calculating the z-transformed correlation coefficients to compare the effective connectivity patterns with those obtained from FC analysis.

Both the significance of effective connectivity parameters and the FC parameters for the optimal model were evaluated by one-sample *t*-test. Correction for FDR was applied to the results with the threshold setting at *p* < 0.05. We further calculated the mean value and standard deviation of the distribution of each effective connectivity parameter across the subjects, as well as of each FC parameter to evaluate the distribution pattern for each connectivity parameter.

### Relationship Among the Connectivity Parameters for All the Pairwise Connections Among the Eight ROIs From FC Analysis, DCM, and GCA

Pairwise correlation coefficients among all the connectivity parameters of the FC analysis, DCM, and GCA at the eight ROIs (the AMY, NAC, VMPFC, bilateral DLPFC, DMPFC, and bilateral insula) were calculated (see **Table 6**).

## Results

### Functional Connections With Brain Areas

**Figure 1:**
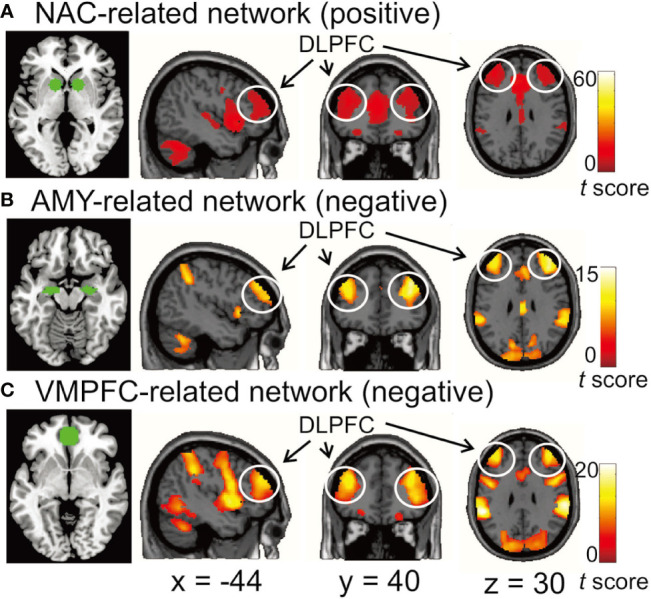
**(A)** the functional network positively correlated with the nucleus accumbens (NAC). **(B, C)** the functional networks negatively correlated with the amygdala (AMY) and ventromedial prefrontal cortex (VMPFC), respectively. The bilateral dorsolateral prefrontal cortex (DLPFC) was functionally connected with all three seed regions. In the left image of each row, the seed regions for functional connectivity analysis are depicted by green. Each network was identified by performing one-sample t-test with a threshold at p < 0.05/3 voxel-wise FWE corrected. Color bars represent *t*-values.

We found the DLPFC to be functionally connected with MDD-related brain areas in distinct ways. The NAC was positively correlated with the bilateral DLPFC, while the AMY and VMPFC were negatively correlated with the bilateral DLPFC (*p* < 0.05/3, voxel-wise FWE-corrected) (**Figure 1**).

**Figure 2:**
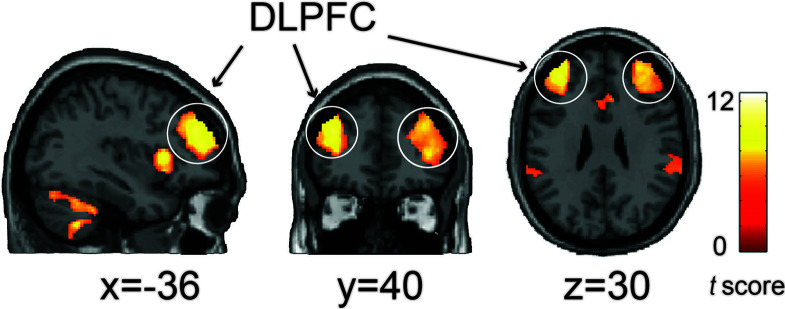
The conjunction analysis showed that the NAC-related network (positive), AMY-related network (negative), and VMPFC-related network (negative) converged on the bilateral DLPFC. Statistical significant threshold was set at *p* < 0.05 voxel-wise FWE corrected. The color bar represents *t*-values.

We also found that these three functionally connected networks overlapped in the bilateral DLPFC (Conjunction analysis: *p* < 0.05, voxel-wise FWE-corrected) (**Figure 2**), and that the bilateral DMPFC, bilateral anterior insula, bilateral IPL and left cerebellum exhibited significant overlap with the three networks (**Table 1**).

**Table 1:** Regions identified by the conjunction analysis of NAC-related network (positive), AMY-related network (negative), and VMPFC-related network (negative).

Identified regions	MNI(mm)			*T* score	Number of voxels
	x	y	z		
Right insula	46	18	2	12.59	1,193
Dorsomedial prefrontal cortex	4	18	46	12.15	776
Left insula	−40	16	4	10.78	590
Left dorsolateral prefrontal cortex	−36	44	28	10.55	1,372
Left cerebellum	−40	−54	−32	8.85	780
Right dorsolateral prefrontal cortex	40	36	34	8.63	1,740
Right superior frontal gyrus	20	6	66	7.91	244
Right inferior parietal lobule	62	−38	46	6.92	793
Left inferior parietal lobule	−56	−40	52	6	358

NAC, nucleus accumbens; AMY, amygdala; VMPFC, ventromedial prefrontal cortex; MNI, Montreal Neurological Institute. Threshold for statistical significance was set at p < 0.05, voxel-wise family-wise error (FWE) corrected. Clusters no less than 100 voxels are listed.

The GM signal scaling shifted the mean values of the correlation coefficients across subjects mostly toward the negative direction (**Tables 2** and **3**). Although most of the negative correlations were already apparent prior to global signal scaling, the correlation coefficients between the DLPFC and amygdala without global signal scaling were still negative but close to zero (**Table 3**).

**Table 2:** Descriptive statistics of pairwise correlation coefficients among the BOLD time series of the eight ROIs (the AMY, NAC, VMPFC, bilateral DLPFC, DMPFC, and bilateral insula) with GM scaling.

	Amy	NAC	VMPFC	lDLPFC	rDLPFC	DMPFC	Linsula	Rinsula
Amy	–	0.0777* ± 0.121	0.300* ± 0.171	−0.175* ± 0.143	−0.202* ± 0.127	−0.144* ± 0.103	−0.110* ± 0.0967	−0.141* ± 0.101
NAC		–	0.0433* ± 0.136	0.158* ± 0.156	0.128* ± 0.139	0.158* ± 0.0971	0.143* ± 0.0951	0.131* ± 0.102
VMPFC			–	−0.315* ± 0.147	−0.238* ± 0.219	−0.228* ± 0.125	−0.255* ± 0.129	−0.279* ± 0.143
lDLPFC				–	0.513* ± 0.339	0.298* ± 0.144	0.321* ± 0.135	0.281* ± 0.129
rDLPFC					–	0.264* ± 0.163	0.157* ± 0.155	0.229* ± 0.162
DMPFC						–	0.404* ± 0.160	0.401* ± 0.212
Linsula							–	0.440* ± 0.157
Rinsula								–

ROI, region of interest; AMY, amygdala; NAC, nucleus accumbens; VMPFC, ventromedial prefrontal cortex; DLPFC, dorsolateral prefrontal cortex; DMPFC, dorsomedial prefrontal cortex; GM, gray matter.

The mean value and the standard deviation of functional connectivity parameters with GM scaling were shown using Z scores. Statistics of the lower triangular part were omitted because they were symmetrical. The diagonal elements were infinite. The parameters shown in * are significantly different from zero (p < 0.05, FDR corrected).

**Table 3:** Descriptive statistics of pairwise correlation coefficients among the BOLD time series of the eight ROIs (the AMY, NAC, VMPFC, bilateral DLPFC, DMPFC, and bilateral insula) without GM scaling.

	Amy	NAC	VMPFC	lDLPFC	rDLPFC	DMPFC	Linsula	Rinsula
Amy	–	0.213* ± 0.196	0.412* ± 0.211	−0.0558* ± 0.203	-0.0687* ± 0.191	0.0239 ± 0.171	0.0113 ± 0.157	−0.00930 ± 0.147
NAC		–	0.159* ± 0.211	0.300* ± 0.189	0.278* ± 0.177	0.289* ± 0.146	0.243* ± 0.134	0.237* ± 0.136
VMPFC			–	−0.244* ± 0.298	−0.136* ± 0.290	−0.0363 ± 0.204	−0.117* ± 0.212	−0.129* ± 0.216
lDLPFC				–	0.695* ± 0.348	0.438* ± 0.156	0.443* ± 0.142	0.410* ± 0.142
rDLPFC					–	0.427* ± 0.155	0.297* ± 0.159	0.373* ± 0.167
DMPFC						–	0.578* ± 0.190	0.582* ± 0.244
Linsula							–	0.577* ± 0.192
Rinsula								–

ROI, region of interest; AMY, amygdala; NAC, nucleus accumbens; VMPFC, ventromedial prefrontal cortex; DLPFC, dorsolateral prefrontal cortex; DMPFC, dorsomedial prefrontal cortex; GM, gray matter.

The mean value and the standard deviation of functional connectivity parameters without GM scaling were shown using Z scores. Statistics of the lower triangular part were omitted because they were symmetrical. The diagonal elements were infinite. The parameters shown in * are significantly different from zero (p < 0.05, FDR corrected).

### The GCA

As shown in **Table 4**, at least more than 73% and 80% of all subjects have significant “Granger causality” for all the pairwise connections and significant causalities in 52 out of the 56 total connections, respectively (**Table 4**).

**Table 4:** Descriptive statistics of pairwise effective connectivity from GCA among the eight ROIs (the AMY, NAC, VMPFC, bilateral DLPFC, DMPFC, and bilateral insula).

	Amy	NAC	VMPFC	lDLPFC	rDLPFC	DMPFC	Linsula	Rinsula
Amy	–	0.0038 (72)	0.0125 (73)	0.0033 (74)	0.0034 (74)	0.0040 (75)	0.0038 (74)	0.0037 (76)
NAC	0.0038 (77)	–	0.0071 (78)	0.0034 (79)	0.0031 (75)	0.0053 (72)	0.0044 (80)	0.0038 (81)
VMPFC	0.0052 (82)	0.0029 (83)	–	0.0051 (80)	0.0044 (79)	0.0033 (84)	0.0042 (79)	0.0045 (85)
lDLPFC	0.0026 (83)	0.0026 (86)	0.0072 (82)	–	0.0103 (82)	0.0091 (87)	0.0113 (78)	0.0047 (85)
rDLPFC	0.0030 (74)	0.0025 (81)	0.0064 (88)	0.0103 (89)	–	0.0125 (90)	0.0040 (91)	0.0072 (87)
DMPFC	0.0030 (92)	0.0037 (77)	0.0049 (80)	0.0084 (90)	0.0089 (88)	–	0.0140 (93)	0.0107 (89)
Linsula	0.0031 (83)	0.0034 (81)	0.0066 (90)	0.0116 (73)	0.0047 (82)	0.0147 (73)	–	0.0158 (94)
Rinsula	0.0031 (95)	0.0026 (92)	0.0076 (88)	0.005 (80)	0.0079 (93)	0.0148 (90)	0.0196 (73)	–

The table shows the descriptive statistics of effective connectivity parameters from GCA for all connections among the psychopathologically depression-related areas (AMY, NAC, and VMPFC), bilateral DLPFC, DMPFC, and bilateral insula. For each connection, the mean value of Granger causality parameters across the subjects were shown. The number of subjects who have the significant Granger causalities is shown in the bracket. Rows and columns correspond to the sources and the destinations, respectively.

### The DCM Analysis

*Post hoc* selection revealed that the fully connected model was the best fitting model. Consistent with GCA, all pairwise connections showed significant “Granger causality”. The distribution of each effective connectivity parameter of the fully connected model across subjects and the FC parameter were shown in **Table 5**. The parameter values were significantly different from zero for most of the pairwise FC parameters within the interested networks (**Tables 2** and **3**), and for 42 out of 64 effective connectivity parameters (*p* < 0.05, FDR corrected) (**Table 5**). For the effective connectivity parameters between the rTMS stimulation sites (left/right DLPFC, DMPFC, and left/right insula) and the psychopathologically MDD-related areas (AMY, NAC, and VMPFC), most of the parameter values were significantly different from zero from the latter to the former, but not from the former to the latter. These results suggest that the information flows between the two sites may primarily be explained by the effective connectivity from the MDD-related areas to the rTMS stimulation sites. However, while the information flows from the NAC to the bilateral DLPFC and DMPFC were not significantly different from zero, it was significantly different from zero from the left insula to AMY and NAC (**Table 5**).

**Table 5:** Descriptive statistics of pairwise effective connectivity from DCM among the eight ROIs (the AMY, NAC, VMPFC, bilateral DLPFC, DMPFC, and bilateral insula).

	Amy	NAC	VMPFC	lDLPFC	rDLPFC	DMPFC	Linsula	Rinsula
Amy	0.0302 ± 0.340	0.0719* ± 0.290	0.0439 ± 0.207	0.0316 ± 0.188	0.0597* ± 0.184	0.0200 ± 0.175	0.0647* ± 0.198	0.0072 ± 0.189
NAC	−0.0683* ± 0.301	0.110* ± 0.343	0.0182 ± 0.188	−0.0032 ± 0.169	−0.0181 ± 0.180	0.0459* ± 0.200	0.0750* ± 0.223	0.0275 ± 0.217
VMPFC	0.308* ± 0.412	0.0702 ± 0.328	−0.198* ± 0.293	0.00 ± 0.187	0.0199 ± 0.209	0.0113 ± 0.186	−0.0060 ± 0.247	0.0055 ± 0.234
lDLPFC	−0.194* ± 0.286	−0.0312 ± 0.289	−0.152* ± 0.226	0.162* ± 0.280	0.0438 ± 0.228	0.117* ± 0.194	0.159* ± 0.216	0.0447 ± 0.226
rDLPFC	−0.217* ± 0.268	−0.0228 ± 0.274	−0.0839* ± 0.214	0.0527* ± 0.229	0.123* ± 0.256	0.180* ± 0.199	−0.0221 ± 0.255	0.119* ± 0.255
DMPFC	−0.436* ± 0.363	−0.0842 ± 0.400	−0.0883* ± 0.300	−0.0850* ± 0.276	−0.132* ± 0.230	−0.190* ± 0.341	0.199* ± 0.341	0.117* ± 0.308
Linsula	−0.442* ± 0.368	−0.133* ± 0.327	−0.213* ± 0.276	−0.102* ± 0.259	−0.133* ± 0.259	0.191* ± 0.239	−0.0678 ± 0.343	0.0883* ± 0.315
Rinsula	−0.368* ± 0.326	−0.108* ± 0.351	−0.165* ± 0.213	−0.0767* ± 0.217	−0.0960* ± 0.191	0.141* ± 0.207	0.204* ± 0.212	0.110* ± 0.321

The table shows the descriptive statistics of effective connectivity parameters from DCM for all connections for the fully connected model among the psychopathologically depression-related areas (AMY, NAC, and VMPFC) and bilateral DLPFC, DMPFC, and bilateral insula. For each connection, the mean value and the standard deviation of connectivity parameter of its distribution were shown. The mean value and the standard deviation of effective parameters were shown in Hz. Rows and columns correspond to the sources and the destinations respectively. The parameters shown in * are significantly different from zero (p < 0.05, FDR corrected).

### Relationship Among the Connectivity Among the Eight ROIs From FC Analysis, DCM, and GCA

Lastly, we examined similarity among functional and effective connectivity parameters. For each connectivity parameter, we first calculated across-subject mean value, then the pairwise correlation coefficients between across-subject means of connectivity parameters derived from the FC analysis, DCM and GCA. As shown in **Table 6**, the correlation coefficients between the FC analysis and GCA and that between the FC analysis and DCM were both larger than that between GCA and DCM. With these results, FC parameters rather than DCM and GCA may be seen as more representative measures to assess the connectivity among the eight ROIs.

**Table 6:** Relationship among the connectivity among the eight ROIs from FC analysis, DCM and GCA.

	FC	DCM	GCA
FC	–	0.49476554*	0.64076112*
DCM	–	–	0.47623865*
GCA	–	–	–

Pairwise correlation coefficients between connectivity vectors which consist of 56 mean connectivity parameters across the whole subjects for pairwise connections among the eight ROIs (the AMY, NAC, VMPFC, and bilateral DLPFC, DMPFC, and bilateral insula) from FC analysis, DCM, and GCA are shown. The correlation coefficients shown in * are significantly different from zero (p < 0.05, FDR corrected).

## Discussion

In the current study, we investigated the functional interactions of MDD-related brain areas using resting-state fMRI data to give a new insight into the underlying neural mechanisms of rTMS treatment for MDD. Three psychopathologically MDD-related brain areas, namely, nucleus accumbens, amygdala, VMPFC, were our main focuses. We found that these three networks converged at one of the effective sites of rTMS treatment for MDD, specifically the bilateral DLPFC (86, 96). Other overlapping brain areas discovered included the DMPFC, bilateral insula and left cerebellum, which could potentially be the new targets for rTMS treatment. The DCM analysis and GCA revealed that there were significant pairwise connections among all psychopathologically MDD-related regions, bilateral DLPFC, DMPFC, and bilateral insula, which implied causal information flow between these areas. The DCM also showed that the psychopathologically MDD-related regions were predominantly feeding information to the rTMS stimulation sites.

### Functional Networks Between Psychopathologically MDD-Related Regions and the rTMS Stimulation Sites

The hypothesis that rTMS-induced neural activities would propagate to other functionally connected distal locations, possibly mediated by polysynaptic neural transmission, has been supported by the findings from studies of MRI (91, 92, 95, 97, 98), electronic stimulation (74, 83, 99), and rTMS applications on patients with MDD (29, 30, 76, 81). For instance, changes of brain activity in MDD patients were observed not only at the site where TMS was applied, but also in distal areas (29, 30, 76, 81). Furthermore, Fox and colleagues have demonstrated that FC predicts effective sites of deep brain stimulation for the treatment of a vast spectrum of psychiatric and neurological disorders (31, 32, 77). Therefore, analysis of FC could be of help in understanding the mechanism of rTMS treatment for MDD. In addition, it could also be used to identify potential new targets of rTMS treatment for various psychiatric disorders.

The idea of the current study is inspired by the findings reported by previous researchers who attempted to link psychiatric illness with brain functions and circuits (33, 34). For instance, the activity of the NAC, highly associated with reward processing, was found to decrease in MDD subjects (17, 58, 75). Other studies found that normalization of AMY-related activities, related to emotion and anxiety, lead to successful interventions for depression (79, 84, 85). It has also been shown that the lesion of the VMPFC, associated with decision making, has the protective effects against depressed mood (19, 80).

The convergence of the FC map suggested that rTMS at the DLPFC would influence the brain areas associated with MDD. Presumably, we could speculate that rTMS treatment improves MDD by normalising the imbalance of regional activity within the MDD symptoms–related regions, as well as the abnormal FC between these brain regions in a synergetic way. Taken together, we proposed that the combination of remote effects on MDD-related networks may explain the treatment mechanism of rTMS on the DLPFC for MDD. Furthermore, the three FC maps also converged on the DMPFC and bilateral insula, suggesting these two sites as potential new targets for rTMS in MDD. For the DMPFC, several studies have already shown that rTMS application over it was effective in improving MDD symptoms (13, 72, 82, 88). With regard to insula, the potential of it as the target for rTMS in MDD had also been confirmed by Philip et al., who found that changes of insula connectivity pattern estimated by multivoxel pattern analysis (78, 87) were associated with clinical improvement of MDD (29). These studies reported findings consistent with our results, which support the notion that the DMPFC and bilateral insula could be new potential targets for rTMS treatment in MDD.

Previous studies have reported that the response rates of rTMS treatments for MDD are moderate (3, 90, 93). We could assume that the rTMS treatment is only effective in certain subtypes of MDD but not the others. This claim is supported by several studies which found abberant brain connectivity patterns associated with the treatment response to rTMS in MDD patients (30, 73, 89, 94). In addition, one recent resting-state fMRI study found that certain subtypes of MDD were more responsive to the rTMS treatment than the others (88).

As DCM and GCA could capture the directional connectivity information that cannot be identified by FC analysis (43, 44, 100, 101), combining these three techniques could better predict the subject-specific responsiveness to rTMS treatment. Since GCA is advantageous to handle a model that constitutes large numbers of sources for regions, we used GCA to narrow down the model space for DCM (65). GCA showed most subjects had significant Granger causality for all of the pairwise connections among the ROIs, and then, we estimated the fully connected model by DCM. DCM also revealed that the fully connected model was the best-fitting model using the *post hoc* BMS, consistent with GCA results. DCM analysis applied to the fully connected model showed that most of the interactions between the psychopathologically MDD-related areas and the rTMS stimulation sites tended to be predominantly explained by the effective connectivity from the former to the latter. This suggested that rTMS treatment could be more effective for patients who have both stronger FC between—and stronger effective connectivity from—the psychopathologically MDD-related areas and to the rTMS stimulation sites than those who have the weaker connectivity for both. However, while there was significant effective connectivity from the NAC to the bilateral insula, no significant information flow from the NAC to bilateral DLPFC or to the DMPFC was found. This suggested that rTMS treatment over insula might be more effective than over the DLPFC or DMPFC for NAC-related symptoms (loss of interest, lack of energy, etc.). Similarly, the significant effective connectivity from the right DLPFC to the AMY and that from the DMPFC to the NAC also implied that rTMS over the right DLPFC and DMPFC might be more effective for AMY-related symptoms (anxiety) and NAC-related symptoms (loss of interest, lack of energy), respectively. Interestingly, only the left insula showed such significant bidirectional effective connectivity with the AMY and NAC. Therefore, the left insula could be a more potent stimulation site for treating the above symtpoms. In addition, the FC analysis revealed that the VMPFC was more strongly connected with the right than left DLPFC. Thus, rTMS treatment on the right DLPFC could be more effective for VMPFC-related symptoms, such as the inhibition of thought process. All considered, we believed that the interaction patterns predict the patient-specific responsiveness to rTMS treatment, which could be the key to optimize a patient-specific rTMS treatment in the future.

In the current practice of rTMS treatment for MDD, the DLPFC is the most widely used target with support on its efficacy to reduce MDD symptoms. It makes sense to believe that the remote effects of rTMS are spread from the DLPFC to the psychopathologically MDD-related areas. Unexpectedly, the causal information we found was quite the opposite. One possible explanation for our finding is that TMS increases the sensitivity of DLPFC in receiving input from the psychopathologically MDD-related areas. Another possibility is that TMS induces both orthodromic and antidromic neural transmissions: One directs impulse to the axon terminals while another one moves the oppositee way toward the somas. The former neural signal could transmit to the psychopathologically MDD-related areas along the connectivity pathway predicted by the DCM model, and strengthen the information flows from the pcyshophysiologically MDD-related areas to the DLPFC, resulting in the remote effects which improve MDD symptoms.

### The Representative Measure Among FC, GCA, and DCM Analysis

The correlations between FC analysis with GCA and DCM are greater than GCA with DCM (**Table 6**). This suggested that FC is the most representative measure among FC analysis, DCM analysis, and GCA. On the other hand, the patterns of the effective connectivity parameters estimated by GCA and by the DCM are different. For example, the ratio of NAC-left DLPFC to left DLPFC-NAC in DCM was relatively large (9.75), while the ratio in GCA analysis was close to 1 (0.76), which suggested that the DCM is more capable of capturing the asymmetric flow of effective connectivity. Furthermore, the number of subjects who had statistically significant effective connectivity originating from the NAC was on average the lowest among all the ROIs of GCA, while most of the effective connectivities projecting to the VMPFC were not statistically different from zero. These results appeared to show that GCA and DCM are not only convergent but complementary to each other (65, 102). This could be caused by the different basic assumptions of the two methods (102). While DCM assesses the change of hidden neural activity from the observed BOLD time series, GCA directly evaluates how the present state of one region statistically relies on the past state of another region (65). In contrast, FC does not require any additional assumptions of effective connectivity. Thus, a FC measure would be a more representative measure than the effective connectivity measures, although effective connectivity can provide additional information flow within the neural networks.

### Convergence of Psychopathologically MDD-Related Networks at DMPFC, Insula, IPL, and Left Cerebellum

In addition to the bilateral DLPFC, we found that the three MDD-related networks converged at the bilateral DMPFC, bilateral insula, bilateral IPL, and left cerebellum. The bilateral DMPFC and insula have been proposed as the new potential targets for rTMS treatment of MDD (13, 29). Sheline and colleagues found that patients with MDD had stronger FC between the DMPFC and the three MDD-related networks, compared to HCs (103). Practically speaking, several studies have shown that rTMS over the DMPFC is an effective method for the treatment of MDD (72, 82). DMPFC is connected to the subgenual cingulate gyrus (SCG) (104), which can be used for predicting rTMS treatment response (30, 105) and is involved in emotion regulation (106, 107). These literatures implied that DMPFC rTMS might be more potent to improve the emotion-regulating network and its related symptoms than DLPFC rTMS. Additionally, Drysdale and colleagues have shown that MDD patients with particular patterns of FC responded to the DMPFC rTMS treatment effectively (88). Previous studies together with our findings of FC convergence appeared to support the usefulness of rTMS treatment on the DMPFC.

The FC convergence can be used as a tool to identify new potential targets of rTMS for other psychiatric disorders, as conceptualized by the previous research linking FC to brain stimulation sites across diverse psychiatric disorders (77). Philip and colleagues found that insula connectivity changes between pre- and post- TMS treatment over left DLPFC were associated with MDD improvement (29). This suggested that rTMS over left DLPFC might improve MDD *via* insula. Anterior insula plays an important role in processing social and affective information (108) and its dysfunction can be commonly seen on MDD patients (109, 110). The anterior insula also constituted the salience network (SN), involved in switching between the DMN and central executive network (CEN) (111, 112). Patients with MDD are impaired in switching between the DMN and CEN, perhaps due to the DMN hyperactivity, and such impairment has been suggested as a key mechanism of the preoccupation with self-referential processes of MDD (110, 113, 114). Thus, direct stimulation of the insula might improve such impairment of MDD.

In our current study, we also found that the three networks converged at the IPL. Several studies have reported abnormalities of IPL for MDD such as decreased metabolism (115), increased regional homogeneity using resting-state fMRI (116), and decreased magnetization transfer ratio (117). The dysregulation of IPL is known to associate with MDD (118).

The convergence also appeared at the left cerebellum. The cerebellum is functionally connected with the frontal and limbic regions with a critical role in emotional and cognitive processing (119, 120). It has been shown that the MDD patients had hypoactive cerebellum in response to emotional stimuli and decreased FC (121, 122). Collectively, our current findings give additional accounts for the roles of the insula, IPL, and cerebellum for MDD.

### Time Scales of the rTMS Effects

While several weeks were necessary to achieve the effect of rTMS treatment, most of the existing TMS-fMRI studies only addressed the immediate response of TMS on brain activity on a time scale between several seconds and an hour. Dowdle and colleagues found that there were immediate increase of neural activities at the ACC, caudate, and thalamus after applying the single-pulse TMS on the left DLPFC (123). Vink and colleagues also found that TMS at the left DLPFC could trigger a strong signal at the connected brain regions, including subgenual ACC, in a concurrent TMS-fMRI experiment (124). Several studies demonstrated functional changes several minutes after 10-Hz rTMS was applied on the left DLPFC. Findings from these studies included an increased cerebral blood flow at the ACC (125), a modulation of dopamine release at the ACC and orbitofrontal cortex (126), and increased connectivity between the left DLPFC and the ACC (27). With such coherent findings, we believed that the hour-long rTMS effects can indeed be extrapolated from the immediate effects. With an even longer time scale, Liston et al. showed that a 5-week course of 10-Hz rTMS over the left DLPFC could modulate several functional interactions. For example, it decreased the hyperconnectivity between the subgenial cingulate cortex and default mode network (DMN) and increases the anticorrelation between the DLPFC and DMN (30). With difficulties to justify the research ethic on applying rTMS intervention to healthy subjects, most long scale data is limited to patients with MDD. However, we are still able to spot some discrepancies between short-term rTMS effects (up to an hour) and long-term rTMS effects (more than several weeks). For example, while rTMS increased the connectivity between the left DLPFC and DMN after 15 min (27), it decreased their connectivity after a 5-week course (30). Although the effect of rTMS on FC is well-supported, how it would lead to either enhancement or suppression remains unclear.

### Limitation

There are several limitations in the present study. First, our current results were derived from the fMRI data of HC instead of MDD patients. The alteration in MDD associated networks was not addressed. Second, instead of directly investigating the rTMS effects on resting-state FC, we inferred their influences from intrinsic functional interactions of brain networks. However, previous studies have demonstrated that FC analysis of resting-state fMRI data without TMS application is adequate to determine an optimal stimulation site of rTMS treatment for MDD (31, 32). In line with these studies, the current study provides extra insights into the potential mechanism of rTMS treatment for MDD. Third, it is still under debate whether regressing out of global signal to calculate the FC is an appropriate practice (127, 128). As shown in **Table 2**, most of the negative correlations from the data with global signal regression would still exist without global signal scaling. We did regress out the average bold signal of GM to perform the FC analysis which was similar to what Fox and colleagues did to calculate the anti-correlated FC between the left DLPFC and the subgenual cingulate (31). In addition, Power and colleagues have shown that global signal regression is necessary if we wished to fully remove the residual artefacts associated with head motion (129). Therefore, the global signal regression would provide a less contaminated estimation of the FC.

### Conclusion

Our FC analysis revealed that psychopathologically MDD-related brain areas converged at the bilateral DLPFC as well as the DMPFC, bilateral insula, and other potential treatment targets. The DCM revealed that most of functional interactions between the psychopathologically MDD-related areas and the DLPFC, DMPFC, and bilateral insula could be primarily explained by effective connectivity from the psychopathologically MDD-related areas to the rTMS stimulation sites, which was not able to be identified by correlation-based FC analysis. The combination of functional and effective connectivity in the MDD-related networks would benefit the prediction of the subject-specific response to rTMS treatment.

In addition, FC parameter is the most robust connectivity parameter among other connectivity parameters from FC, GCA, and DCM. We argued that the convergence of the functional brain networks related to a psychiatric disorder could underpin the potential targets of the brain stimulation treatment and its mechanism through remote modulatory effects.

## Data Availability Statement

The original contributions presented in the study are included in the article/supplementary material; further inquiries can be directed to the corresponding author.

## Author Contributions

Conceived and designed the study: YM. Analyzed the data: TI and YM. Wrote the paper: TI, TD, WS, and YM.

## Funding

This study was supported by JSPS KAKENHI (Grant Number JP18K15491; TI) and by the Japan Science and Technology Agency PRESTO program (10238; YM).

## Conflict of Interest

The authors declare that the research was conducted in the absence of any commercial or financial relationships that could be construed as a potential conflict of interest.

The handling editor declared a shared affiliation with one of the authors, TI, at time of review.
